# What are the functional outcomes and service experiences of patients with head and neck cancer treated during the COVID-19 pandemic?

**DOI:** 10.1007/s00520-024-08811-w

**Published:** 2024-08-29

**Authors:** Sarah Day, Kira Mabb, Jodie Nixon, Jocelyn Williames, Mair Emlyn-Jones, Kate Davis, Christie Barrett, Laurelie Wishart, Bena Brown

**Affiliations:** 1 Nutrition and Dietetics Department, Princess Alexandra Hospital, Brisbane, Australia; 2 Consumer Partnering, Metro South Health Clinical Governance, Risk and Legal, Brisbane, Australia; 3 Occupational Therapy Department, Princess Alexandra Hospital, Brisbane, Australia; 4 Physiotherapy Department, Princess Alexandra Hospital, Brisbane, Australia; 5 Radiation Oncology, Princess Alexandra Hospital, Brisbane, Australia; 6 Social Work Department, Princess Alexandra Hospital, Brisbane, Australia; 7 Centre for Functioning and Health Research, Metro South Health, Brisbane, Australia; 8 School of Health and Rehabilitation Services, The University of Queensland, Brisbane, Australia; 9 Southern Queensland Centre for Excellence in Aboriginal and Torres Strait Islander Health Care, Metro South Health, Brisbane, Australia; 10 School of Public Health, University of Queensland, Brisbane, Australia

**Keywords:** Head and neck cancer, COVID-19, Radiotherapy, Functional outcomes, Allied health

## Abstract

**Introduction:**

Head and neck cancer (HNC) care was significantly impacted by the COVID-19 pandemic. The current study aimed to explore the functional outcomes and service experiences of patients with HNC treated during and prior the COVID-19 pandemic.

**Methods:**

Mixed methods were used to (1) retrospectively compare HNC patients’ functional outcomes and allied health service usage across two time-controlled cohorts and (2) understand the experiences of HNC care using validated surveys and qualitative interviews.

**Results:**

Retrospective data was extracted for 78 participants (pre-COVID-19, *n* = 43; during-COVID-19, *n* = 35), with *n* = 28 and *n* = 18 completing surveys and an interview, respectively. Significant differences were found in service modality between groups with significantly more phone and telehealth services provided during COVID-19. Service usage and functional outcomes were not significantly different between groups. During-COVID-19 participants reported being significantly less informed about their care and care was significantly less appropriate and acceptable. Thematic analysis of interviews revealed six broad themes related to communication, person-centred care, treatment logistics, care availability, safety of care, and impacts on experiences.

**Conclusions:**

This study revealed that whilst HNC care rapidly changed at the onset of COVID-19, patient access to treatment and functional outcomes did not differ significantly. Rather, factors related to the patient experience of care were discussed. Healthcare professionals working in HNC have further evidence supporting building relationships based on transparent communication and partnering with patients to overcome rapid clinical changes, as experienced during COVID-19.

**Supplementary Information:**

The online version contains supplementary material available at 10.1007/s00520-024-08811-w.

## Introduction

The COVID-19 pandemic impacted healthcare delivery in unprecedented ways. Strategies were rapidly adopted to reduce transmission risk, including physical distancing, telephone and telehealth care, mask mandates, and hospital visitor restrictions. Head and neck cancer (HNC) treatment during the COVID-19 pandemic posed risk of viral exposure to healthcare workers [1] and increased psychological distress to patients and carers due to distancing/isolation measures [2]. Furthermore, fewer patients presented for investigation during COVID-19, and those who did present had a higher proportion of T3/4 tumours [3].

HNC and the required treatment has significant impacts on an individual’s physical functioning and psychological well-being [4, 5]. Treatments are complex and require multidisciplinary (MDT) care in the acute phase, recovery, and survivorship [6, 7]. This includes integration of individual patient’s needs, perspectives, and preferences, to ensure person-centred care [8]. Prior to COVID-19, usual care for people with HNC treated with radiotherapy at the study site included face-to-face services from medical, nursing, and allied health in accordance with best-practice guidelines [6]. However, rapid practice change was required during COVID-19 to safely deliver treatment in line with optimal care pathways. Service delivery was affected through hospital resource redistribution, transition to telehealth modalities, reduction of face-to-face services, and the inability to conduct routine examinations due to aerosolising procedure risks [9]. It is unknown whether this rapid practice change impacted on functional outcomes (anthropometrical changes, oral intake and alternative feeding, the presence of pain, fatigue, lymphoedema, and level of distress) or on patient perspectives of the care they received.

Several studies have investigated the impact of COVID-19 on HNC treatment, reflecting the complexities of providing timely patient care while maintaining clinician and patient safety [1, 3, 10]. However, few studies have been published in the Australian context or explored the patient experience of HNC treatment during COVID-19. The current study aimed to explore HNC service usage, patient functional outcomes, and patient perspectives of outpatient service delivery, prior to and during the COVID-19 pandemic.

## Methods

### Study design

This study used mixed methodology over two phases, at a tertiary cancer centre in Australia. Phase One retrospectively compared functional outcomes and service metrics of people with HNC across two time-controlled cohorts: “pre-COVID-19” (April–July 2019) and “during COVID-19” (April–July 2020). Following informed consent, Phase Two utilised mixed methods, through validated surveys and a qualitative semi-structured interview to explore participant experiences of HNC care during the same time periods. The qualitative component of this study was underpinned by phenomenology with the intention to describe the experiences of people with HNC accessing health services prior to and during COVID-19 [11].

### Recruitment

For Phase One, participants were included if they were ≥ 18 years, diagnosed with HNC of any stage (oral cavity, oropharynx, larynx, hypopharynx, nasopharynx, salivary gland, or metastatic skin to parotid/neck) and completed outpatient curative intent treatment (post-operative or definitive radiotherapy with or without chemotherapy) at the study site during the specified time periods. Eligible participants underwent standard care and received healthcare services from medical, nursing, and one or more allied health disciplines during their treatment. Participants were excluded if they did not receive (or complete) curative intent treatment, were palliative, or received treatment for recurrent disease. Participants were not excluded on the basis of primary language spoken or any psychosocial factors.

Participants identified as eligible from Phase One were invited to participate in Phase Two by completing patient experience surveys and a 30-min interview. Recruitment occurred between July and December 2021. Participants who declined, were unable to be contacted, or had confirmed cognitive deficits, end-stage disease, and/or undergoing palliative management were excluded from Phase Two.

### Procedure

For Phase One, data was extracted for eligible participants through a retrospective audit of the electronic medical record (ieMR) and scheduling system. Data collected included participant demographics, diagnostic details, allied health service metrics, hospital admission data, and radiation treatment replans. Available functional outcomes were collected from participant medical charts at pre-determined timepoints, providing these were documented in routine clinical practice reporting: baseline (from date of treatment plan to within 2 weeks of first radiation treatment), treatment completion (within 2 weeks of final radiation treatment), and post-treatment (within 2–3 months post final radiation treatment). Functional outcome data included anthropometrical measures (weight, percentage weight change, body mass index [BMI]), alternative feeding (tube type, prophylactic or reactive insertion), the presence of pain, fatigue, lymphoedema, Functional Oral Intake Scale (FOIS) [12], and distress using the distress thermometer [13].

In Phase Two, surveys and interviews were completed via phone or face-to-face, depending on participant preference. Participants completed four validated surveys: (1) the Australian Hospital Patient Experience Question Set (AHPEQS) [14]; (2) Acceptability of Intervention Measure (AIM); (3) Intervention Appropriateness Measure (IAM); and (4) Feasibility of Intervention Measure (FIM) [15]. Following survey completion, participants completed a semi-structured interview. Interviews included open-ended questions to explore participants’ experience of HNC care and impacts of COVID-19 on the care received. Questions related to care by allied health were probed to understand specifically these experiences of care. A participant interview guide was provided to the participant by the interviewer, to aid reflection of care experiences. Participants were welcomed to have a support person or carer present, who was able to participate in the interview if desired.

All interviews were conducted by a single interviewer, not previously known to the participants, or involved in their cancer care. The interviewer was a female, experienced clinical accredited practicing dietitian at the study centre and introduced herself to participants as such. The interviewer received qualitative interview training from a senior researcher and utilised the interview guide to optimise interview consistency. The interview guide was designed based on expert clinical knowledge, current literature, and to elucidate experiences of care. The interview guide was piloted on five participants, with minor changes to question order and wording made. Participants did not provide feedback on the interview guide. All interviews were audio-recorded on a research specific Apple smartphone using the Voice Record Pro application (4.0.3 build 4031-p216, running on iOS17.5.1), de-identified and transcribed verbatim. Participants were offered the opportunity to review their interview transcript to amend content.

### Data analysis

In Phase One, descriptive statistics (means, median, frequencies, standard deviations [SD]) were used to analyse participant characteristics for both the pre-COVID-19 and during-COVID-19 cohorts. Parametric (*t*-test, chi-square) or non-parametric statistics (Fishers’ exact, Mann–Whitney *U*) were used to determine differences between group variables.

In Phase Two, survey data was collapsed as recommended by the AHPEQS and AIM/IAM/FIM authors into binary classifications [14, 15]. Non-parametric statistics (Fisher’s exact) were used to determine differences between cohort responses due to small sample size and cell counts. Interview transcripts were analysed by three members of the research team using the 6-phase inductive approach, described by Braun and Clark [16]. To begin, all three members of the research team independently immersed themselves in the data, reading, re-reading the transcripts, and making initial notes to familiarise themselves with the dataset. Initial codes were generated across the dataset, independently by the three members of the research team analysing the dataset. Data relevant to the initial codes were collated by each of the three team members. Following this, two members of the research team used their initial codes to code three interview transcripts together. A preliminary coding tree was developed following this stage that reflected the consensus decision of the codes between the two research team members. The third team member then joined an additional consensus meeting to further develop the coding tree. Following this, each of the three team members coded the transcripts assigned to them (the principal investigator coded all interview transcripts and randomly assigned each transcript to one of two HNC clinical research experts for coding). Codes were collated into potential themes, and all data coded under these potential themes independently by each of the three team members. The three team members then met again to review the assignment of codes to themes and define and name the themes, categories, and subcategories emerging from the dataset. Coding disagreements were discussed amongst the three research team members, reflecting on the intention of the participant, context, and nature of the coded data. After collaborative discussion, consensus was reached for all coding disagreements. Themes, categories, and subcategories were determined using an iterative approach, with the three team members sharing their independently generated themes, which were then grouped, collapsed, or expanded as a result of discussion between the team members. The final step to decide on compelling exemplar data examples to form the basis of this report was determined by the principal investigator.

## Results

**Fig. 1:**
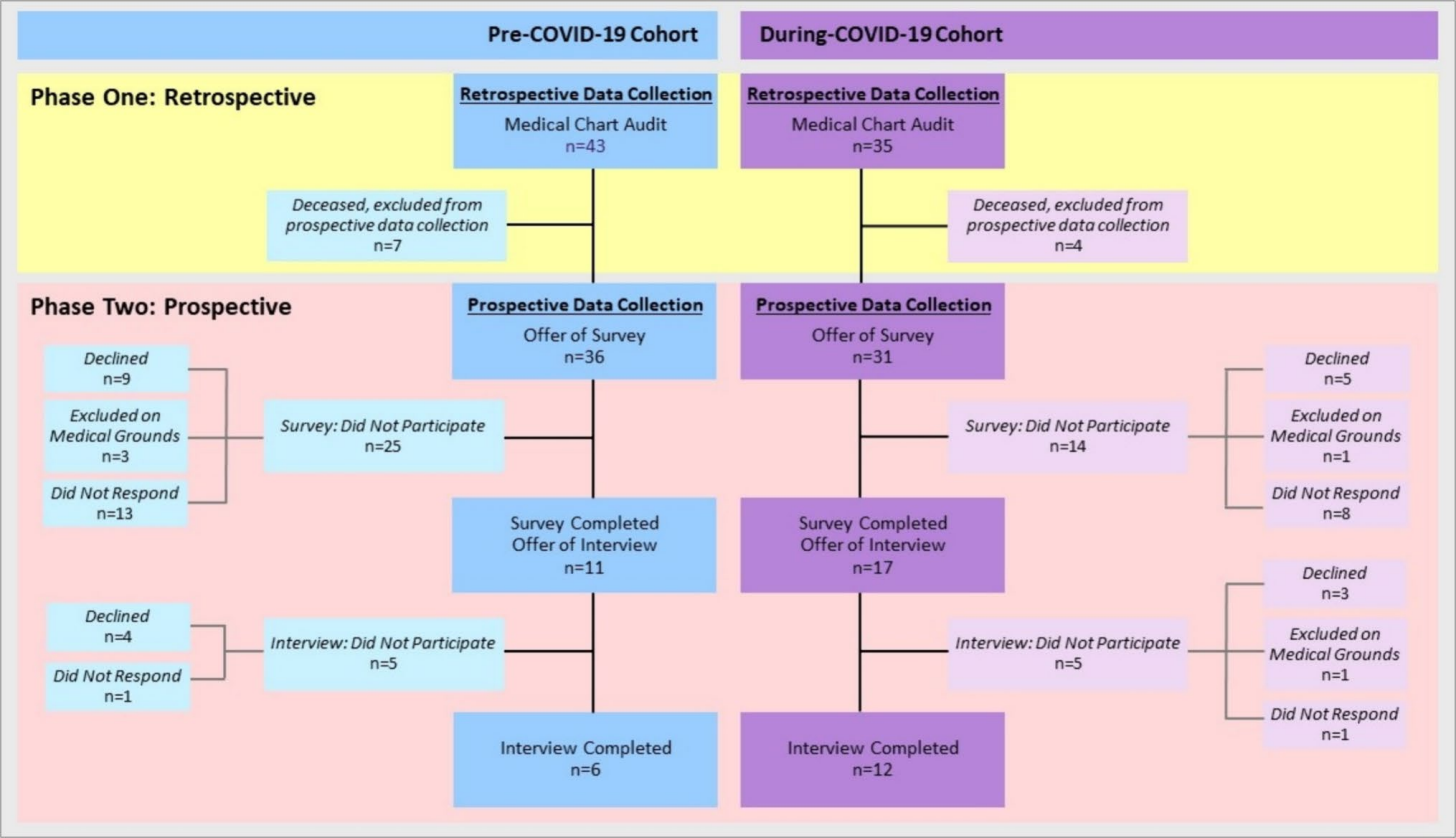
Recruitment for surveys and interviews of pre-COVID-19 and during-COVID-19 cohorts

Ethical clearance was received from the local Human Research Ethics Committee (HREC/2021/QMS/72421) to conduct this project. Phase One retrospective data was extracted for eligible participants from the pre-COVID-19 group (*n* = 43) and the during-COVID-19 group (*n* = 35). Participants were male (*n* = 64, 82.1%), in their early 1960s, received chemoradiotherapy (*n* = 45, 57.7%) for oropharyngeal cancers (*n* = 38, 48.7%), and had a support person on treatment (*n* = 61, 78.2%). There were no significant differences between groups (Table 1). Of the 78 participants from Phase One, 67 (85.9%) participants were eligible for Phase Two surveys and interviews, with 28 (41.8%) participants completing the surveys and 18 (26.9%) participants completing interviews (Fig. 1).

**Table 1:** Patient demographics and functional outcomes

	**Pre-COVID-19** ***N* = 43**		**During-COVID-19** ***N* = 35**		**t/chi**^**2**^	***P***
	N	(%)	N	(%)		
**Age, years** Mean (range)	62.8 (46–85)		64.5 (37–85)		0.337	0.563
**Gender** Male Female	35 8	(81.4) (18.6)	29 6	(82.9) (17.1)	0.02	0.888
**Tumour site** Oral cavity Oropharynx Hypopharynx/larynx Nasopharynx Skin/neck	5 24 5 4 5	(11.6) (55.8) (11.6) (9.3) (11.6)	4 14 5 3 9	(11.4) (40.0) (14.3) (8.6) (25.7)	N/A†	0.863
**Clinical stage** Early (stage 1–2) Late (stage 3–4)	21 22	(48.8) (51.2)	11 24	(31.4) (68.6)	1.75	0.186
**Treatment modality** RT^a^ CRT^b^ Surgery + RT Surgery + CRT	6 27 8 2	(14.0) (62.8) (18.6) (4.7)	4 18 12 1	(11.4) (51.4) (34.3) (2.9)	N/A†	0.487
**Support person on treatment** Yes No	30 13	(69.8) (30.2)	31 4	(88.6) (11.4)	2.98	0.084
**SEIFA index‡** Median (range)	6 (1–10)		6 (1–10)		N/A§	0.689
**Distress**^**c**^** at baseline** Yes (score ≥ 4) No (score ≤ 3) Missing data	12 30 1			27.9% 69.8% 2.3%	13 21 1	37.1% 60.0% 2.9%
**FOIS**^**d**^** at baseline** Full/soft (score 6 or 7) Modified (score 4 or 5) Tube fed (score 1, 2, or 3) Missing data	33 7 2 1			76.7% 16.3% 4.7% 2.3%	32 1 0 2	91.4% 2.9% 0.0% 5.7%
**FOIS at Tx**^**e**^** completion** Full/soft (score 6 or 7) Modified (score 4 or 5) Tube fed (score 1, 2, or 3) Missing data	9 13 19 2			20.9% 30.2% 44.2% 4.7%	12 11 10 2	34.3% 31.4% 28.6% 5.7%
**Feeding tube on Tx** Yes No	22 21			51.2% 48.8%	13 22	37.1% 62.9%
**Feeding tube type** NGT^f^ RIG^g^	17 5			77.3% 22.7%	8 5	61.5% 38.5%
**Insertion timing** Prophylactic (pre-tx) Reactive (on-tx)	4 18			18.2% 81.8%	5 8	38.5% 61.5%
**Feeding tube in use** End of Tx 3 months post tx	19 3			86.4% 13.6%	11 3	84.6% 23.1%
**Height, cm**^**h**^ Mean (SD^i^)	*N* = 42 174 cm (9.3)		*N* = 35 176 cm (8.9)		0.96	0.341
**Weight, kg**^**j**^** at baseline** Mean (SD)	*N* = 43 79.9 kg (15.8)		*N* = 35 88.2 kg (23.1)		1.88	0.064
**Weight, kg at Tx completion** Mean (SD)	*N* = 43 75.9 kg (14.7)		*N* = 34 84.5 kg (22.1)		2.04	0.044
**BMI**^**k**^**, kg/m**^**2l**^** at baseline** Mean (SD)	*N* = 42 26.4 kg/m^2^ (4.9)		*N* = 34 28.3 kg/m^2^ (6.4)		1.47	0.147
**BMI, kg/m**^**2**^** at completion** Mean (SD)	*N* = 42 25.1 kg/m^2^ (4.5)		*N* = 34 27.1 kg/m^2^ (6.0)		1.66	0.101
**%**^**m**^** Weight loss Tx completion** Mean (SD)	*N* = 43 − 5.5% (5.6)		*N* = 34 − 4.7% (4.9)		0.66	0.513
**% Weight loss 3 mths**^**n**^** post Tx** Mean (SD)	*N* = 36 − 11.5% (7.6)		*N* = 23 − 9.7% (6.8)		0.92	0.360
**Fatigue at Tx completion** Yes Not reported	25 18		58.1% 41.9%		22 13	62.9% 37.1%
**Lymphoedema 3 months post Tx** Yes Not reported	10 33		23.3% 76.7%		9 26	25.7% 74.3%
**ED**^**o**^** and/or hospital admission on Tx** Patient admitted on treatment * Yes—one admission* * Yes—multiple admissions* * No*	16 10 17		37.2% 23.3% 39.5%		11 5 19	31.4% 14.3% 54.3%
Total admissions per cohort * Non-treatment related* * Treatment-related*	39 3 36		7.7% 92.3%		21 3 18	14.3% 85.7%

^†^Fisher’s exact test performed

^‡^SEIFA index: Postal Area (POA) Index of Relative Socio-economic Advantage and Disadvantage, 2016

^§^Mann–Whitney *U* test performed

^*^p value =  < 0.05

^a^Radiotherapy; ^b^chemoradiotherapy; ^c^as measured by Distress Thermometer [13]; ^d^Functional Oral Intake Scale [12]; ^e^treatment; ^f^nasogastric tube; ^g^radiographically inserted gastrostomy; ^h^centimetres; ^i^standard deviation; ^j^kilogram; ^k^body mass index; ^l^metres squared; ^m^percentage; ^n^months; ^o^emergency department

### Phase One: impact of COVID-19 on functional outcomes

There were no significant differences in functional outcomes between groups, except the during-COVID-19 group had higher body weight at the conclusion of treatment (*p* = *0.044*) (Table 1). Despite this finding, there was no significant difference in percentage weight loss between groups (*p* = *0.513*) and the during-COVID-19 group trended toward higher body weight at baseline (*p* = *0.064*), although this was not significant. There were no differences in emergency and/or hospital admissions between groups. Radiation treatment replans only occurred for two patients per cohort.

### Phase One: impact of COVID-19 on allied health service delivery

Due to the introduction of phone and telehealth during COVID-19, the service modality between groups was statistically significant for all allied health professions (speech pathology, dietetics, occupational therapy, and social work), except physiotherapy which trended towards significance (*p* = *0.05)* (Table 2).

**Table 2:** Allied health occasions of service (OOS)

Appointment modality by profession	Pre-COVID-19 *N* = 43		During-COVID-19 *N* = 35		Fishers Exact	
	Total OOS^c^ (*N*)	Average/pt	Total OOS (*N*)	Average/pt	*p*	
Joint Appointment: Speech Pathology (SP) & Dietitian (DN)					< 0.001*	
F2F^a^	257	5.98	113	3.32		
Phone	0	0	61	1.74		
VTC^b^	0	0	22	0.63		
Individual appointment: SP or DN					< 0.001*	
F2F	76	1.77	11	0.31		
Phone	0	0	3	0.09		
VTC	0	0	1	0.03		
Physiotherapy (PT)					0.050	
F2F	33	0.77	27	0.77		
Phone	0	0	4	0.11		
VTC	0	0	0	0		
Occupational therapy (OT)					< 0.001*	
F2F	66	1.53	38	1.09		
Phone	0	0	16	0.46		
VTC	0	0	2	0.06		
Social work (SW)					< 0.001*	
F2F	78	1.81	4	0.11		
Phone	0	0	51	1.46		
VTC	0	0	0	0		
Total—Allied Health					< 0.001*	
F2F	526	12.23	194	5.54		
Phone	0	0	135	3.86		
VTC	0	0	30	0.86		
Total occasions of service	Total OOS (*N*)	Average/pt^d^ (SD)	Total OOS (*N*)	Average/pt (SD)	T test	
					*t*	*p*
SPDN joint	257	5.98 (2.25)	196	5.6 (2.05)	0.78	0.443
SP or DN individual	76	1.77 (1.54)	15	2.33 (0.45)	− 2.07	0.041
PT	33	0.77 (1.39)	31	0.89 (1.39)	− 0.38	0.706
OT	66	1.53 (1.89)	56	1.60 (2.21)	− 0.15	0.881
SW	78	1.81 (2.11)	55	1.57 (1.63)	0.55	0.583
Total—Allied Health	526	12.23 (5.18)	359	10.26 (4.91)	1.71	0.091

^*^p value =  < 0.05

^a^Face-to-face; ^b^videoteleconference; ^c^occasions of service; ^d^patient

For the overall number of appointments, the during-COVID-19 cohort receiving significantly less individual speech pathology or dietetic appointments (*p* = *0.041*) (Table 2). Otherwise, there was no significant difference between groups for the number of appointments received.

### Phase Two: impact of COVID-19 on health service experience

#### Surveys

Using the AHPEQS, the during-COVID-19 group reported being significantly less (*p* = *0.049*) informed about their care compared with the pre-COVID-19 group, with no other significant differences between groups (Table 3). On AIM/IAM/FIM surveys, the during-COVID-19 group reported their care was significantly less appropriate and less acceptable by being less: fitting (*p* = *0.012*), suitable (*p* = *0.012*), applicable (*p* = *0.049*), appealing (*p* = *0.018*), or a good match (*p* = *0.018*), when compared with the pre-COVID-19 group (Table 3).

**Table 3:** Survey results

Survey and questions	Pre-COVID-19, *n* = 11				During-COVID-19, *n* = 17				
**Australian Hospital Patient Experience Question Set (AHPEQS)**	“*Always*” “*Very Good*”		All other responses		“*Always*” “*Very Good*”		All other responses		***p***
	*N*	(%)	*N*	(%)	*N*	(%)	*N*	(%)	
*My views and concerns were listened to*	10	90.9%	1	9.1%	12	70.6%	5	29.4%	0.355
*My individual needs were met*	9	81.8%	2	18.2%	9	52.9%	8	47.1%	0.226
*I felt cared for*	10	90.9%	1	9.1%	11	64.7%	6	35.3%	0.191
*I was involved as much as I wanted in decision making*	7	63.6%	4	36.4%	6	35.3%	11	64.7%	0.246
*I was kept informed as much as I wanted*	10	90.9%	1	9.1%	9	52.9%	8	47.1%	**0.049**
*The staff involved in my care communicated with each other†*	7	63.6%	4	36.4%	11	68.7%	5	31.3%	1.000
*I received pain relief that met my needs***‡**	7	77.8%	2	22.2%	8	47.1%	6	35.3%	0.400
*I felt confident in the safety of my treatment and care*	10	90.9%	1	9.1%	11	64.7%	6	35.3%	0.191
*Overall, the quality of treatment and care I received was*	11	100%	0	0.0%	13	76.5%	4	23.5%	0.132
**Survey of experience of care during HNC**^**a**^** treatment**	“*Completely Agree*”		All other responses		*“Completely Agree”*		All other responses		***p***
	*N*	(%)	*N*	(%)	*N*	(%)	*N*	(%)	
**Acceptability of Intervention Measure (AIM)** *The care I received during my HNC treatment…*									
*Met my approval*	11	100%	0	0.0%	11	64.7%	6	35.3%	0.055
*Was appealing to me§*	9	90.0%	1	10.0%	7	41.2%	10	58.8%	0.018*
*I liked the care I received§*	9	90.0%	1	10.0%	11	64.7%	6	35.3%	0.204
*I welcomed the care I received*	10	90.9%	1	9.1%	11	64.7%	6	35.3%	0.191
**Intervention Appropriateness Measure (IAM)** *The care I received during my HNC treatment…*									
*Seemed fitting§*	10	100%	0	0.0%	9	52.9%	8	47.1%	0.012*
*Seemed suitable§*	10	100%	0	0.0%	9	52.9%	8	47.1%	0.012*
*Seemed applicable*	10	90.9%	1	9.1%	9	52.9%	8	47.1%	0.049*
*Seemed like a good match for me§*	9	90.0%	1	10.0%	7	41.2%	10	58.8%	0.018*
**Feasibility of Intervention Measure (FIM)** *The care I received during my HNC treatment seemed implementable*	9	81.8%	2	18.2%	12	70.6%	5	29.4%	0.668

^*^p value =  < 0.05

^†^*n* = 16 during-COVID-19; 1, non-response; ‡, excluded from analysis as reported “didn’t apply” (*n* = 2 pre-COVID-19; *n* = 3 during-COVID-19), §, *n* = 10 pre-COVID-19; 1, non-response

^a^Head and neck cancer

#### Interviews

**Fig. 2:**
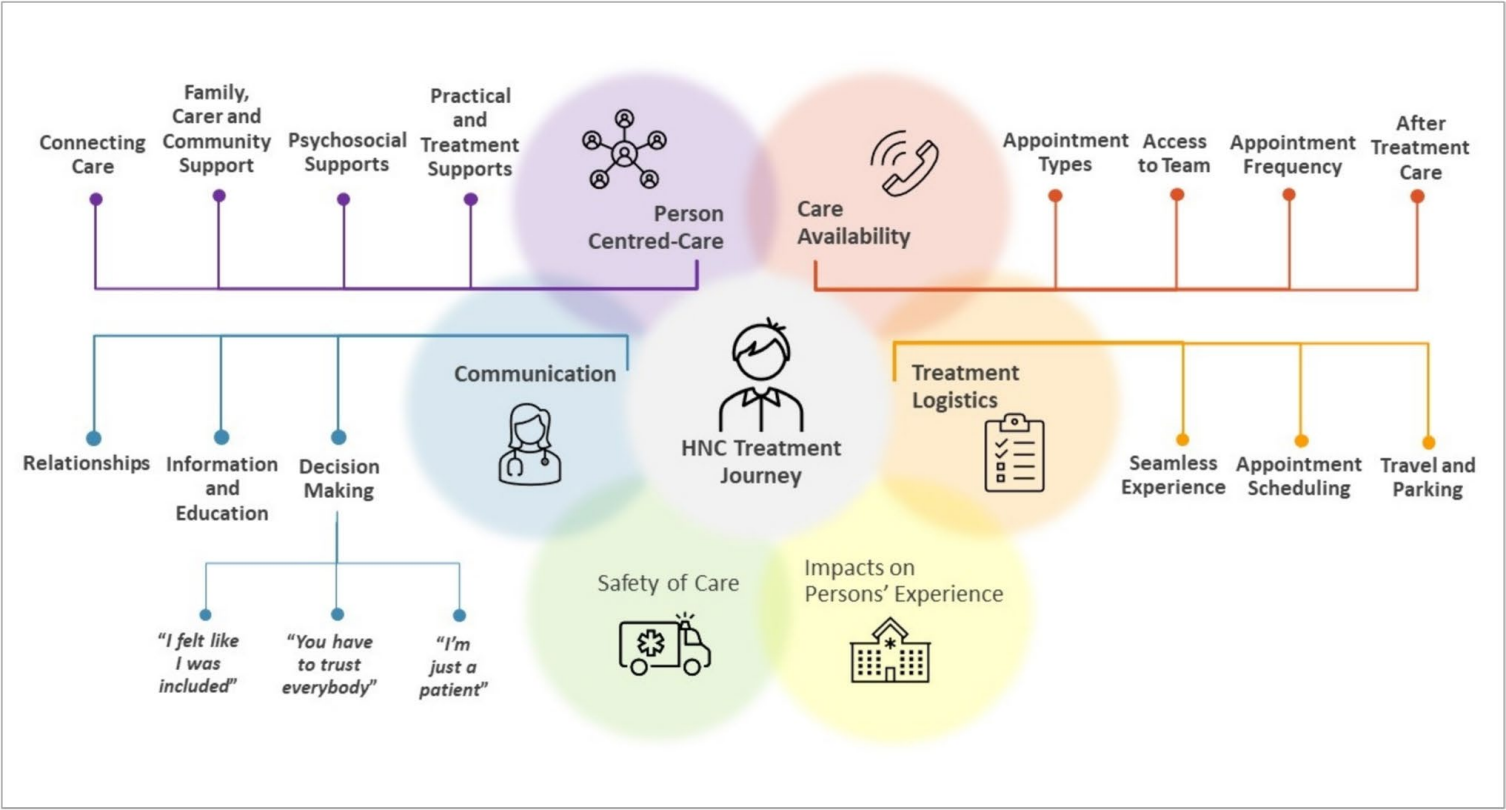
Thematic analysis of themes, categories, and sub-categories

Majority of interviews were conducted via phone (*n* = 16, 88.9%), with two being face-to-face. Interview duration ranged from 13 to 37 min (mean = 23 min) for 17 of the 18 interviews, with one interview spanning 8 min. Two participants had a support person present and participating in the interview. Thematic analysis revealed six broad themes describing participants’ experiences of the HNC care received (Fig. 2): communication, person-centred care, treatment logistics, care availability, safety of care, and impacts on persons’ experiences. Participants in both groups discussed their experiences of HNC care across these six themes, with exemplar quotes summarised in Table 4.

**Table 4:** Themes, categories, and sub-categories with exemplar quotes

Theme	Category	Sub-category and supporting qualitative data	
**Communication**	Decision Making	“I felt like I was included”	“I felt listened to, definitely” (9B) “If there’s options, they always let you know… So it was always in the conversation” (13B)
		“You have to trust everybody”	“We put our trust in them, and we think it was justified” (21B) “I just kept doing what they said… So the overall thing, I thought I was well looked after” (35B)
		“I’m just a patient”	“I don’t want to be known as a bad patient by having questions…” (14B) “They were just trying to force bloody milkshakes down me… They just kept going on and on” (26B)
	Information and Education	“The staff were really good, also the doctors… giving me explanation of what’s going on and just helping you along…” (27B) “They were all good, and explained everything really well before I set off in the course of treatment…” (17A)	
	Relationships	“The professionalism and the compassion shown… was fantastic, it was way beyond what I imagined it would be…” (9B) “It was as if they were almost family members” (34A)	
**Person-centred care**	Practical and Treatment Support	“And diet wise, that was very helpful, with my diet and putting me onto certain supplements to take” (17B) “The occupational therapist was good, they gave me the exercises and materials to do at home” (17A)	
	Psychosocial Support	"The nurses were friendly and helpful and they put up with my problems. I suffer from PTSD from the war… when they like put a tube in me throat, to breathe, and just my PTSD come back after being gone for many years" (15B) “There was a lady who was like a support when I was doing the chemotherapy… she was I thought, excellent and very good to have someone like that sort of on the side, but over the top watching what everybody was doing” (19A)	
	Family, Carer, Community Support	“I have a great network of friends and family, and I sort of had gathered them before I started…” (14B) “I have two brothers who would’ve gone out of their way for me, and I thank them. I just said, if I need you, I will call” (34A)	
	Connecting Care	“It didn’t matter what time of day with the nurses changing over, or even doctors, they still knew who you were at all times” (27A) “I would have liked maybe to be scheduled in when the same staff that I was always used to see me again… it became a personal thing” (34A)	
**Treatment logistics**	Seamless Experience	“It was all pretty programmed” (9B) “I just felt the whole procedure was really well managed” (17B)	
	Appointment Scheduling	“When they say they’re going to call at a certain time on a certain day, they do… you can set your clock by it” (21B) “I understand that the doctors are busy and all that. But sometimes you wait for an hour and a half to see them” (32B)	
	Travel and Parking	“I really appreciated having the parking” (17B) “Towards the end [of treatment] … you’re just not that cognitive… You’re the last person that should be getting in a taxi or on a bloody public transport” (7A)	
**Care availability**	Appointment Types	“I prefer face-to-face [appointments] myself” (35B) “I did prefer either tele or zoom… I'm over an hour away, so it was far better for me physically and from a time perspective” (14B)	
	Access to Team	“They said, if you need any assistance just give us a call” (33B) “We did call outside the schedule several times and they did answer” (21B)	
	Appointment Frequency	“The dietitian I probably saw maybe two or three times during those three weeks and I thought it was adequate” (11A) “I think I would have liked to have seen the ENT guys a bit more” (35B)	
	After Treatment Care	“I had less communication after I was finished. I think I said, ‘look I’m fine, I don’t need’ [follow-up]” (13B) “The follow up it’s been a bit of a joke which I find a bit weird, because I’m pretty aware that this isn’t a cheap treatment” (7A)	
**Safety of care**		“At first it [COVID] was scary, cause you didn’t know what to expect, but once we were in there, we were right” (9B) “The canula was the one that I though oh God, are they slipping up a little bit on some routines? Did I feel unsafe? At the time probably not…” (19A)	
**Impacts on persons’ experience**		“I’m not a looking back person. I would like to look forward, you know… like forget it and move on” (15B) “I don’t think there was any option not to go ahead with the operation… that was my best chance” (17B) “I’ve still got a lot of chemo fog. Yeah, I just sort of really sort of can’t remember things” (27B)	

##### *Theme 1: Communication*

“Communication” was a major theme, describing interactions with their healthcare team under three primary categories. The “decision making” category revealed how participants and healthcare teams interacted to make decisions. Participants discussed three sub-categories demonstrating a range of experiences. The first sub-category “I felt like I was included” reflected partnering in healthcare decisions: “I was supported, and I was made felt that if I wanted to contribute, they didn’t…discard me as garbage… I did not feel that I was excluded” (34A). Through sub-category 2 “You have to trust everybody”, participants reflected experiences of being confident in health professionals to led decision-making, while still feeling involved in their care decisions: “I went along with what the team said, because that was obviously the best way out of the situation” (9B). The third sub-category, “I’m just a patient”, reflected experiences of being excluded from decision making: “…like I said they did something completely different, and yeah, I realised I wasn’t a partner, I’m just a patient” (15B). In category 2, “information and education”, most participants reflected information provision/education was satisfactory: “The discussions gave me all the information that made me satisfied that I was getting the right treatment” (11A). Category 3, “relationships”, was described positively by most participants regarding the personalised care received: “She called [him] by name every time… that gives you a personal touch, which is very important” (21B-carer).

##### *Theme 2: Person-centred care*

Participants discussed “person-centred care” under four categories (Table 4), emphasising the importance of healthcare staff integrating their needs, preferences, and values and the significance of being treated as a whole person.

“Practical and treatment support” (category 1) reflected positive experiences of healthcare staff addressing participants day-to-day needs: “allied health were fantastic, followed up and everything” (33B) and “the nurses in the oncology after I had my radiation treatment were bloody terrific” (35B). For category 2, “psychosocial support”, participants had mixed reflections. One felt their psychosocial needs were unmet, necessitating care external to the health service: “I had a trauma therapist that I had been working with since my diagnosis… I felt it was the only part of the team that wasn’t addressed” (14B). In contrast, others felt positively regarding the psychosocial support received: “I was extremely aggressive… and for people to be able to see past that and read into the subtext of this ‘oh, this person really wants help and he’s actually grateful’, is astounding” (7A). “Family, carer and community support” (category 3) was integral to most participants HNC experience. Strong reflections emerged regarding participant support networks: “…having him [my husband] there in that early stage everyday… it was really important” (17B). “Connecting care” (category 4) was less prominent, where participants discussed the value of ongoing connections with familiar health professionals across the phases of the treatment (Table 4).

##### *Theme 3:Treatment logistic*s

Participants reflected their experience within the health system, including their appointment coordination through treatment, as reported in theme 3 “treatment logistics”. While most found the health system efficient, others experienced delays and disruptions. Overall, majority of participants summarised their care coordination as a “seamless experience” (category 1). Value was placed on the ease of the process: “everything just seemed to go like clockwork” (1B). In category 2 “appointment scheduling”, most participants discussed positive experiences of organisation. “You never sat for very long waiting. It was very well co-ordinated” (13B). However, another found wait times spanned longer: “there were more people than staff could handle” (34A). “Travel and parking” (category 3) contained mixed experiences. Those who received ambulance transport were positive: “the patient transport scheme… it was very smooth” (35B). In contrast, one participant elaborated travel to and from the hospital as challenging (Table 4).

##### *Theme 4: Care availability*

“Care availability” was a major theme discussed by participants, and those treated during the COVID-19 pandemic were required to engage with the health team in new and dynamic ways. As such, most discussion reported here was experienced by the during-COVID-19 cohort accessing allied healthcare, with four primary categories emerging (Table 4). The “appointment types” category revealed how participants felt about face-to-face, phone, and telehealth service delivery models. Participants reflected strong preference for face-to-face care: “I still believe you cannot… get the same results as face-to-face… even on Zoom you can’t see the body language” (27B). Participants discussed interventions delivered via phone with mixed experiences. Some found the convenience advantageous: “I preferred to have the phone [appointments] because I’m so far away” (13B), whereas others reflected reduced engagement: “I just didn’t used to answer my phone. I knew it was them ringing” (26B). Regarding telehealth, participants spoke positively of convenience, but found the technology challenging: “I had one telecall thing with the dietitians and the rest were by phone, which suited me better to go by phone than that bloody computer I can tell you” (35B). Participants spoke highly of the availability to engage with health professionals on an as-needs basis, through category 2 “access to team”. “I had a booklet with all the phone numbers and contact people and I could have just rung at any time” (17A). Category 3, “appointment frequency”, was a highly variable experience. While some reflected appointment frequency met their needs “I was quite happy with the frequency of what I saw, who I saw and when I saw them” (33B), others felt appointments were too frequent “I felt quite overwhelmed with the amount of time that I needed to spend with them” (14B). When participants reflected on the post-treatment phase of their HNC journey, under category 4 “after treatment care”, the overwhelming experience was navigating recovery without healthcare support: “the patient after treatment is sort of like let go… But meanwhile you have to live with this on a daily basis and that’s hard…” (21B).

##### *Theme 5: Safety of care*

“Safety of care” emerged as a less prominent theme, where participants discussed staffing levels, experience and expertise, and the impact COVID-19 had on feeling safe in the hospital environment. A large proportion felt very satisfied with care safety, expressing minimal impact of COVID-19; “I can’t recall an occasion where I didn’t feel safe” (9B). Where safety concerns were expressed, this primarily related to COVID-19 exposure risk in the hospital setting: “I wasn’t touching the handrails anywhere there. I’m thinking everyone that’s got COVID is coming down here” (1B).

##### *Theme 6: Impacts on persons’ experience*

The sixth theme, “impacts on persons’ experience”, emerged through participants discussing how their HNC treatment affected them physically and psychologically, in the short-term and through post-treatment recovery. Many reflected on the psychological impact of treatment, their coping strategies, resilience, and determination to get through treatment: “I was there to fight, get on with it, get well and get out of there” (7A). When asked to reflect on the impact of COVID-19 on their cancer care experience, most described it as “a non-event” (13B). Requirements of surgical masks and increased hand hygiene was met with little concern: “I’m an old soldier. I’m used to being told what to do by people, so I’ve just adjusted and went with the flow” (15B). Conversely, others were impacted by physical distancing and hospital entry restrictions: “My partner cannot go in… but sometimes I need my partner to be with me because my English is not perfect and maybe I will miss something” (21B).

## Discussion

The current study explored the differences between two cohorts of people receiving treatment for HNC, prior to and during the COVID-19 pandemic. While similar in demographic profiles and service usage, as expected, those receiving treatment during COVID-19 received significantly more telephone/telehealth interventions. They also felt significantly less informed of their care and found their care significantly less acceptable and appropriate compared with their pre-COVID-19 counterparts. Deeper exploration through qualitative interviews reflected themes related to the experiences of HNC care, relating to the importance of communication, person-centred care, logistics, care availability, and safety of care.

COVID-19 presented cancer centres with significant challenges to rapidly change standard care and minimise transmission risk. Previous studies have reported implementation of mask mandates, social distancing, minimising visitor interactions, and moving healthcare interactions to phone or telehealth [17, 18] as risk mitigation strategies. Within HNC, the pandemic impact included diagnostic delays [17], increased tumour burden [3], and concerns regarding impacts on cancer care and recovery [19]. Investigating diagnostic delay, palliative intent treatment, and medical appointments and investigations was not within the scope of the current study. For our cohort undergoing HNC treatment with curative intent, we found minimal difference in patients’ access to healthcare (allied health and nursing) and no difference in functional outcomes. These positive outcomes found in the current study may be a result of the rapid transition to alternative models of care (telephone/telehealth) that did not interrupt access to care and the expertise of the clinical team being able to rapidly pivot towards alternatives than minimise COVID-19 transmission. These strategies reduced face-to-face contact and minimised the use/frequency of aerosolising procedures, telehealth swallow assessments, and for those requiring face-to-face interventions being isolated away from contact with other patients/health professionals. Those receiving treatment during COVID-19 were significantly less informed of their care and found their care significantly less acceptable and appropriate compared with their pre-COVID-19 counterparts. It could be hypothesised that the strategies to reduce COVID-19 transmission, particularly in relation to minimising non-essential, face-to-face contact between patients and their healthcare team also resulted in a reduction of informal conversations and sharing of information about their care. During interviews, participants reported COVID-19 had minimal influence on their experience during treatment. This finding has been substantiated by McAndrew et al. [20] who found patients were more concerned about their cancer than contracting COVID‐19. While some participants discussed the risks of contracting COVID-19 in the hospital environment, this was a minor theme. Our cohort reflected more on COVID-19 risk mitigation policies, including visitor restrictions and challenges of upholding physical distancing in the hospital setting. Across both cohorts, participants emphasised the importance of family, carer, and community support during HNC treatment and the small number who were impacted by visitor restrictions reflected this. Similar experience studies have reported patients being negatively affected by restrictions, through reduced emotional support and difficulties in retaining information [2].

As cancer services’ model of care rapidly adjusted in response to COVID-19, participants in the current study reflected the impact on their experience. Overall, face-to-face remained the preferred method for patients to connect with their treating team and build patient-clinician relationships. However, the advantage of phone communication was acknowledged as being more accessible and time-saving, improving the ease of engagement, and reducing travel. Other participants shared that technology posed challenges and was suboptimal compared to face-to-face contact, as previously reported [21]. Other studies of HNC populations have demonstrated when telehealth is used in appropriate circumstances it is feasible, acceptable, and accessible modality for patient care [22, 23].

Two strong themes discussed by both cohorts in our study were the importance of communication and person-centred care. Overall, both cohorts felt included in their care, but some reflected they could be better engaged in decision-making. A small subset of participants in the during-COVID-19 group had experiences where they felt rushed and dismissed in respect to making treatment decisions. Previous studies have reiterated the need to consider patient preferences and values and to consistently incorporate them at an organisation level [24]. In the cancer context, the HNC optimal care pathway also reiterates this recommendation [6]. Our study found that despite the impact of COVID-19, positive experiences of integrating support networks and family in patient care were strong. Despite this, meeting the individualised psychosocial needs of participants was inconsistent. It is known from pre-pandemic studies that HNC patients are at higher psychosocial risk [25]; therefore, particular effort should be made to meet patient needs.

Participants discussed the level of after treatment support they received from their healthcare team. While some expressed health professional follow-up was not required, it was more commonplace that patients and carers felt isolated in recovery and found adjusting to post-treatment life challenging, irrespective of whether participants received treatment pre-COVID-19 or during-COVID-19. These findings are supported in studies where HNC patients report a wide variety of unmet needs including acute toxicities and longer-term survivorship concerns [26–28].

There are limitations to this study. Phase one was retrospective in nature, and while these results are reflective of data routinely collected in clinical practice, there are limitations including incomplete documentation or missing data points. The small interview sample size, as well as one interview that was quite short (8 min), may impact how representative the reported experiences are of the wider patient cohort, particularly for the pre-COVID-19 group who were difficult to recruit. As expected, due to the time between receiving treatment and completing the interview, recall bias would have a significant impact on reflections shared by participants in experience interviews. Comparing the qualitative pre-COVID-19 results with the during-COVID-19 results presents unknown variables. As participants elected to engage in the study, participation-bias may impact the experiences discussed. Future studies capturing patient experience should be completed in a timely manner to ensure accurate reflections of care experience. Potential alternative models of care that are patient-led and inclusive of experiences and needs throughout may be worthwhile exploring.

## Conclusion

HNC care rapidly changed at the onset of the COVID-19 pandemic; however, patient access to treatment and functional outcomes did not differ in the current study. Rather, factors related to the patient experience of care, including the importance of communication and person-centred care, were reported. Healthcare professionals working in cancer care have further evidence supporting building relationships based on transparent communication and partnering with patients to overcome rapid clinical changes, as experienced during COVID-19. Cancer centres worldwide need to ensure staff are highly skilled, flexible, and focussed on the patient-health professional relationship. Future research could examine patient recommendations or strategies to improve their healthcare experience during time of practice change.

## Supplementary Information

Below is the link to the electronic supplementary material.

Supplementary file1 (PDF 177 KB)

## Data Availability

No datasets were generated or analysed during the current study.
