# Supplementary material for: What are the functional outcomes and service experiences of patients with head and neck cancer treated during the COVID-19 pandemic?
Source: Support Care Cancer. 2024 Aug 29;32(9):620. doi: 10.1007/s00520-024-08811-w (PMC11362178; doi:10.1007/s00520-024-08811-w)
Supplement: Supplementary file 1 — Supplementary file1 (PDF 177 KB) [file 520_2024_8811_MOESM1_ESM.pdf]

Interview Guide – For Patients

Title: What are the functional outcomes and service experiences of patients with head and neck cancer treated during the COVID-19 pandemic?

Short title: Head and neck cancer treatment during COVID-19

Lead researcher: Ms Sarah Day, Dietitian, Princess Alexandra Hospital
Research Team: Dr Bena Brown, Kira Mabb, Dr Jodie Nixon, Jocelyn Williames, Mair Emlyn-Jones, Kate Davis, Christie Barrett, Dr Laurelie Wishart

---

The purpose of this interview is to explore your experiences and perspectives of the supportive care you received during your treatment for head and neck cancer.

Everyone's experiences are different and there are no right or wrong answers. We are interested in hearing about your experiences and perspectives experiences and perspectives of the supportive care you received during your treatment for head and neck cancer. Nothing you say will be traced back to you and you are free to stop at any time without having to give a reason. If there is a question that you don't want to answer you may ask to move on to the next question.

Below is a list of the questions that you may be asked during the interview.
Before the interview you can think about and reflect on your experiences.

Background

- What stands out in your mind about the care you received during your head and neck cancer treatment?
- Tell me about how it felt walking into the hospital for your head and neck cancer treatment

People

- Think about the people that supported you during treatment. That might be family and friends. It also includes the team of people at the hospital.
- What stands out about the people around you during treatment?

PROBES:

- o Which members of the health care team do you remember seeing?
- o Probe other members if they are not mentioned

Services

- Reflect on the appointments with people at the hospital. How did you interact with the team of health professionals?

PROBES:
  - Did you receive appointments face-to-face / over the phone / telehealth?
  - Did you have a preference for the type of appointment?
  - Did you get to choose how you received care?
  - What did you think of face-to-face vs phone vs telehealth appointments?

Frequency
- Think about how often you saw the different members of the health care team. You may have seen the nurses and radiation therapists every day, and the doctors and people in allied health (like the dietitian or physiotherapist) each week.
- Did the frequency of seeing people in your team seem about right?

PROBES:
  - Did this differ for when you were on-treatment vs after treatment completed?
  - Were there any team members you would have preferred to have seen more or less?
  - Did you feel you had access to team members when you needed them if this was outside of your scheduled appointments?

COVID-19 (IF PARTICIPANT RECEIVED TREATMENT BETWEEN April – July 2020)
- Think about the changes that were occurring with the COVID-19 pandemic, in regards to physical distancing, restrictions in support people attending hospital, utilisation of masks and protective equipment.
- How did this impact on your overall experience with treatment?

PROBES:
  - Is there more your healthcare team could have done to support you during this time?

Building a Relationship
- Do you feel like you were included as a partner in your health care decisions?

PROBES:
  - Did you feel listened to?
  - Were your opinions and preferences taken into account?
  - Did the care you received feel safe?

General Questions:
- Is there anything you wish was different in the way you experienced your care?
- Is there anything further you would like to share in relation to your treatment experience?

We look forward to hearing your experiences in the interview.
